# In Silico Decoding of Parkinson’s: Speech & Writing Analysis

**DOI:** 10.3390/jcm13185573

**Published:** 2024-09-20

**Authors:** Robert Radu Ileșan, Sebastian-Aurelian Ștefănigă, Radu Fleșar, Michel Beyer, Elena Ginghină, Ana Sorina Peștean, Martin C. Hirsch, Lăcrămioara Perju-Dumbravă, Paul Faragó

**Affiliations:** 1 Department of Neurology and Pediatric Neurology, Faculty of Medicine, University of Medicine and Pharmacy “Iuliu Hatieganu” Cluj-Napoca, 400012 Cluj-Napoca, Romanialperjud@umfcluj.ro (L.P.-D.); 2 Department of Oral and Maxillofacial Surgery, Lucerne Cantonal Hospital, Spitalstrasse, 6000 Lucerne, Switzerland; 3 Department of Computer Science, Faculty of Mathematics and Computer Science, West University of Timisoara, 300223 Timisoara, Romania; sebastian.stefaniga@e-uvt.ro (S.-A.Ș.); radu.flesar02@e-uvt.ro (R.F.); 4 Medical Additive Manufacturing Research Group (Swiss MAM), Department of Biomedical Engineering, University of Basel, 4123 Allschwil, Switzerland; 5 Department of Anglo-American and German Studies, Faculty of Letters and Arts, “Lucian Blaga” University of Sibiu, 550024 Sibiu, Romania; elena.ginghina@ulbsibiu.ro; 6 Institute for Artificial Intelligence in Medicine, Faculty of Medicine, University Hospital Giessen and Marburg, Philipps-Universität Marburg, Baldingerstraße, 35043 Marburg, Germany; martin.hirsch@uni-marburg.de; 7 Bases of Electronics Department, Faculty of Electronics, Telecommunications and Information Technology, Technical University of Cluj-Napoca, 400114 Cluj-Napoca, Romania; paul.farago@bel.utcluj.ro

**Keywords:** Parkinson’s global challenge, in silico, speech and handwriting biomarkers, decision support systems, artificial intelligence, cognitive automation, smartphone sensors, digital health technologies, socioeconomics, fastest-growing neurodegenerative disease

## Abstract

**Background**: Parkinson’s disease (PD) has transitioned from a rare condition in 1817 to the fastest-growing neurological disorder globally. The significant increase in cases from 2.5 million in 1990 to 6.1 million in 2016, coupled with predictions of a further doubling by 2040, underscores an impending healthcare challenge. This escalation aligns with global demographic shifts, including rising life expectancy and a growing global population. The economic impact, notably in the U.S., reached $51.9 billion in 2017, with projections suggesting a 46% increase by 2037, emphasizing the substantial socio-economic implications for both patients and caregivers. Coupled with a worldwide demand for health workers that is expected to rise to 80 million by 2030, we have fertile ground for a pandemic. **Methods**: Our transdisciplinary research focused on early PD detection through running speech and continuous handwriting analysis, incorporating medical, biomedical engineering, AI, and linguistic expertise. The cohort comprised 30 participants, including 20 PD patients at stages 1–4 on the Hoehn and Yahr scale and 10 healthy controls. We employed advanced AI techniques to analyze correlation plots generated from speech and handwriting features, aiming to identify prodromal PD biomarkers. **Results**: The study revealed distinct speech and handwriting patterns in PD patients compared to controls. Our ParkinsonNet model demonstrated high predictive accuracy, with F1 scores of 95.74% for speech and 96.72% for handwriting analyses. These findings highlight the potential of speech and handwriting as effective early biomarkers for PD. **Conclusions**: The integration of AI as a decision support system in analyzing speech and handwriting presents a promising approach for early PD detection. This methodology not only offers a novel diagnostic tool but also contributes to the broader understanding of PD’s early manifestations. Further research is required to validate these findings in larger, diverse cohorts and to integrate these tools into clinical practice for timely PD pre-diagnosis and management.

## 1. Introduction

### 1.1. Parkinson’s Disease: A Global Challenge

More than two centuries have passed since James Parkinson’s “An Essay on the Shaking Palsy” in 1817, where he detailed a then-rare disease based on six cases [1]. Now recognized as Parkinson’s disease, it has become the fastest-expanding neurological disorder globally [2]. When expressed quantitatively, there is an observable exponential increase in incidence, with the count rising from approximately 2.5 million in 1990 to 6.1 million in 2016 [3]. Predictions indicate an expected doubling in numbers by 2040 [4]. Link these data to global trends in increasing life expectancy and its myriad implications, coupled with a burgeoning global population approaching 8.1 billion, and we find ourselves in fertile ground for a pandemic, as other research has shown [2,4]. The financial dimension follows a parallel trajectory, though challenging to quantify. A thorough study in 2017 revealed that the total economic impact of Parkinson’s disease amounted to $51.9 billion in the U.S. [5]. Furthermore, this study forecasted a 46% surge in total expenses by 2037 [5]. While the numbers are compelling, the broader socio-economic implications of the disease on affected individuals and their caregivers encompass a wide range of issues. These include loneliness and isolation, stigma, employment challenges, mental health concerns, and social withdrawal.

### 1.2. The Importance of Early Recognition: Exploring Prodromal Indicators

F.A. Porsche’s assertion that an understanding of an object’s function often reveals its form [6] is an apt encapsulation of the diagnostic approach to Parkinson’s disease, particularly in its prodromal phase, which can last over 20 years and often eludes detection through conventional symptoms [7,8]. Since 2017, our transdisciplinary research group has been inspired by Ray Kennedy’s early Parkinson’s retrospective assessment [9] to shift from traditional approaches to digital and AI-driven methods. Although our research initially concentrated on gait analysis, we have recently broadened our scope to encompass speech and handwriting. 

Going back to James Parkinson’s 1817 “An Essay on the Shaking Palsy” [1], it is clear that Parkinson himself recorded observations of speech impairment (Cases II and VI) and writing restrictions, noting that the patient: “would perhaps seldom think of his being the subject of disease, except when reminded of it by the unsteadiness of his hand, whilst writing or employing himself in any nicer kind of manipulation”. The status quo places greater emphasis on speech impairment, moving from its recognition as a distinct symptom of progressive PD, characterized by a clinical phase with motor onset, such as soft speech [10], to its potential role as a biomarker for early PD with rapid eye movement disorder, associated with deficits in articulation, phonation, and prosody [11]. Conversely, handwriting deficits or dysgraphia are recognized as two of the first manifestations when motor symptoms affect the dominant hand [12]. In this context, we identified a potential opportunity and a crucial research topic: to investigate speech and handwriting as potential biomarkers for early, subtle manifestations of Parkinson’s disease.

### 1.3. Speech and Handwriting as Key Biomarkers

To develop a Parkinson’s disease decision support system (DSS), we partnered with a linguist to utilize our database compiled from a Romanian-speaking cohort. Romanian’s distinctive blend of native elements, Latin roots, and Slavic and Germanic influences [13]—part of the Indo-European language family, like widely spoken English—positions us to extend our development seamlessly to other Latin, Germanic, and Slavic languages once our approach is proven successful. We believe that it is essential to rigorously investigate all aspects of research, including the intrinsic properties of language, in order to accurately assess speech and handwriting biomarkers.

A comprehensive literature search was conducted using the PubMed, IEEE Xplore, and ScienceDirect databases to identify relevant studies in English, with no temporal limitation placed, on speech or handwriting analysis and artificial intelligence related to Parkinson’s disease (PD). The search yielded 103 papers: 24 on the evidence-based impact of Parkinson’s disease, 9 on metrics for PD evaluation, 58 on communication assessment (6 on linguistics, 33 on speech, and 19 on writing), and 12 on the use of artificial intelligence in pre-diagnosing PD. Criteria for pooling the literature review included study design, sample size, statistical analysis, and consistency with other research, focusing specifically on the qualitative evaluation of speech and handwriting features in Parkinson’s disease. The studies indicated consistent markers for hypokinetic dysarthria in speech analysis, such as reduced loudness, imprecise articulation, and voice tremor. In handwriting analysis, micrographia and motor control issues emerged as common markers.

#### 1.3.1. Speech Analysis: Linguistic Biomarkers

Hypokinetic dysarthria covers a broad spectrum of speech disorders, including phonation, articulation, respiration, resonance, and prosody, which, in the context of PD, manifest in monoloudness, monopitch, imprecise articulation, short utterances, rapid accelerations/decelerations, breathy and hoarse voice, and respiratory deficiencies [14,15,16]. 

Phonatory deficiencies are evaluated through sustained vowel phonation, assessing pitch [17,18,19,20,21], intensity—based on mean, variability, and dynamic range [18,20], formant frequencies [17], phonation tremor—based on jitter and shimmer [22], voice quality—based on the harmonic-to-noise ratio [19,23], and aerodynamic insufficiency [16]. Articulatory decay [17] and improper consonant articulation [24] are evaluated through diadochokinetic tasks (syllable repetition) and voicing of isolated words or short sentences.

The assessment of prosodic elements of continuous speech, through either monologue delivery or passage reading, includes monoloudness, analyzed based on energy features [25], and monopitch, analyzed based on the fundamental frequency [26,27,28] and time-domain periodicity features [25,29]. The assessment of speech nasality is based on the power spectrum, the group delay spectrum, and cepstral coefficients [30,31,32]. Articulation is examined by formant analysis [20,26,28]. Continuous speech assessment also includes voice quality [16] and timing [29], focusing on speech rate and the duration of pauses, thereby offering a comprehensive analysis of speech dynamics.

The speech tasks may be employed alongside spectral features, such as Mel-Frequency Cepstral Coefficients (MFCC) [33,34,35,36] and wavelet transform [37,38], within machine learning frameworks [39,40,41]. Such methodologies facilitate the characterization of the vocal tract, vocal folds, and deviations in the fundamental frequency [16]. 

#### 1.3.2. Handwriting Analysis: Allographic Features

Parkinsonian handwriting exhibits specific manifestations, the most prominent being micrographia, motor planning difficulties and interference from tremor, dystonia, and dyskinesia [42,43]. 

Writing assessments in Parkinson’s disease are performed with various writing tasks [44,45]: graphical elements (e, m, q, s task) [46,47], circle/spiral [47,48], individual letters [49,50], individual words [48], and sentences/text [51]. It is noteworthy to observe the lack of solutions for continuous writing assessment, but rather the extraction of individual letters from sentences via unsupervised clustering [51] or pen-up and pen-down gestures [50]. The writing tasks can be performed on various media, such as paper or tablet [42,47,48,49]—with a clear preference of the subjects for paper [50]— and the task can also be recorded with a camera [48]. 

The appearance of handwriting is mainly characterized in terms of size [47,50,51,52] and slant [51]. Fine motor movements, motor control, coordination, and cognitive functions related to handwriting tasks are assessed with kinematic features: position/coordinates [50,52], velocity [50,52,53], acceleration [42,50,52], and fluency/jerk [46,48,49,52,54]. However, according to [45], kinematic features are highly task-dependent.

Motor control and cognitive functions related to handwriting tasks are assessed in terms of dynamic features: duration [42,51], altitude and azimuth [47,55], tilt [47], and pressure [47,50,53]. Muscular activity during the handwriting task can be assessed with surface EMG [47]. Also of interest is the assessment of in-air movements [42,51]. 

### 1.4. Unifying Artificial Intelligence (AI) and Parkinsonian Communication Deficit Assessment

The revised Movement Disorder Society Research Criteria for Prodromal Parkinson’s disease have been expanded to include new markers and underline the role of continuous, sensor-based quantitative analysis of motor and non-motor symptoms using AI-powered wearables [56]. The use of artificial intelligence technologies in Parkinson’s disease management is poised to support diagnostic accuracy, optimize treatment planning, and raise the standard of patient care [57]. By progressively using AI algorithms to mine both complex and previously under-explored biomedical data, this approach is setting new benchmarks in early detection capabilities, as outlined above. Recent strides in AI have showcased its efficacy in early PD identification through patient sketch analysis using basic-architecture convolutional neural networks (CNNs). Despite their straightforward nature, these CNNs have delivered remarkable outcomes in pinpointing PD patterns, underscoring the value of continued research and its integration within the medical domain [58]. Utilizing a pre-trained model on a targeted dataset, a study reported 86% accuracy in differentiating 10 PD patients from 10 healthy controls [59]. The evaluation’s depth was limited, in our opinion, by the absence of detailed protocol information and the reliance on clearly distinct patterns in the dataset’s images. One study achieved a 96.67% accuracy by applying transfer learning to sketch drawings using a ResNet V50, highlighting the feasibility of attaining substantial results from small datasets [58,60]. Another study employed Extreme Machine Learning (EML) with rapid convergence speed and a newly designed CNN architecture on a 135-spectrogram dataset, which was augmented to 1755 through modifications primarily in the color spectrum [61]. This augmentation approach is debatable, as it essentially reiterates the same information in varied color spectrums.

Despite the growing prevalence of AI algorithms in medical practice, many of them demonstrate convenient metrics for accurate results only in conditions of small datasets, which carry a high risk of overfitting or misinterpretation.

Our transdisciplinary endeavor, founded upon artificial intelligence, was dedicated to the enhancement of the precision of early Parkinson’s disease detection and the fostering of the creation of intelligent decision support and remote monitoring systems. We accomplished this by developing a new CNN architecture, utilizing transparent high-volume data, and leveraging the F1 accuracy score.

### 1.5. Research Aims and Potential Impact 

This research aims to advance the early detection of Parkinson’s disease by using a novel, comprehensive, fully automated approach to analyze raw speech and handwriting data from diagnosed individuals, ultimately creating software that can identify prodromal patterns. Since 2021, our transdisciplinary team, comprising experts in medical, biomedical engineering, AI, and linguistics, has validated a novel protocol for a neurodegenerative decision support system (DSS) called ParkinsonNet. This research employs an in-silico approach to analyze speech and handwriting as potential early biomarkers of Parkinson’s disease. In a clinical research setting designed to mimic real-life scenarios, we conducted a de novo analysis of speech and handwriting characteristics, proposing an innovative correlation-based approach, leading to the assessment of 16 distinct characteristics. Notably, our study uniquely focuses on running speech and continuous writing, in contrast to previous research, which has mainly focused on sustained vowel phonation, diadochokinetic tasks, or word/sentence voicing for speech, and repetition of graphic elements (e.g., e, m, q, s), shapes (circles/spirals), or individual letters for writing assessment. In the case of complex patterns, such as those pertaining to legibility or collective phonetic traits, we have devised combined depictions that are inspired by Apple’s latest system-on-a-chip (SoC) architecture, where each section is tailored for a specific function in a multi-process operation. Similarly, each section of the depiction is assigned to a communication-related manifestation of the disease. The ParkinsonNet model, in conjunction with a 34,860-image-strong dataset, yielded F1 scores of 95.74% for the combined speech and 96.72% for the combined writing datasets.

This study’s findings will pave the way for advanced research into biomarkers for the early diagnosis of neurodegenerative diseases. Promoting open-source and transdisciplinary collaborations enhances this endeavor. The statement, “Parkinson’s Disease: A Global Challenge”, not only defines our research focus but also serves as a call to action. The intention is to open-source the ParkinsonNet DSS model architecture with the objective of evaluating biomarkers in the prodromal phase through multicentric prospective studies. By incorporating DSS and utilizing raw data from common technologies, such as smartphone recordings and handwritten documents, our study has the potential not only to refine early detection methods for Parkinson’s disease, but also to contribute to the future establishment of remote monitoring and management techniques for the field.

The final objective is to develop a free mobile application to monitor disease progression and treatment. This initiative will leverage emerging generative AI technology linked to real-time biosensors to support both consumers and professionals. For instance, the app could generate personalized writing exercise therapies that, once approved by the treating specialist, may significantly improve patient quality of life. Additionally, with a maxillofacial surgeon on our team, we aim to enhance our software to simultaneously assess REM sleep behavior disorder and obstructive sleep apnea, potentially transforming both general prevention and prodromal evaluation. Moreover, the broader impact of our findings could influence various neurodegenerative diseases and medical fields, enhancing human–machine interactions, especially in robotics.

## 2. Materials and Methods

### 2.1. Study Design

This prospective case-control study took place at the Department of Neurology and Pediatric Neurology, University of Medicine and Pharmacy, “Iuliu Hatieganu”, Cluj-Napoca. The study was conducted in Romania, with recruitment and data collection occurring between September 2021 and December 2023. Ethics approval was obtained from the Ethics Committee of the aforementioned university.

### 2.2. Cohort Description

The research cohort, Table 1, comprised 30 participants, including 20 Caucasian patients in medication-ON state, aged 47 to 75 with Parkinson’s disease at stages 1–4 on the Hoehn and Yahr scale, and 10 age-matched Caucasian healthy controls. Dementia was the primary exclusion criterion.

### 2.3. Procedures

The acquisition of speech and writing was conducted under clinical conditions designed to mirror real-life patient interactions, without any special provisions for soundproofing or noise reduction. Our study introduces the novelty of using running speech and continuous writing, distinguishing it from previous research focused on segmented tasks. For the speech task, participants read a given text aloud at a natural speed. The readings were recorded with a 44.1 kHz sampling rate and saved as MPEG-4 files. For the handwriting task, participants were directed to transcribe dictated text onto a blank A4 paper using a ballpoint pen. In the context of our study, we considered various media options for the writing tasks, including paper, tablets, and video recording. We ultimately chose paper to facilitate the dissemination of our software in underdeveloped regions, with the intention of easily transitioning to a digital approach later. Subsequently, the handwritten samples were scanned with 600 dpi and transferred to a computer as JPEG files. These images were then converted to monochrome, with additional adjustments made to luminosity and contrast.

We adhered to the MDS-UPDRS evaluation guidelines for speech and writing. Skorvanek et al. indicates that MDS-UPDRS is suitable for assessing mild to moderate symptoms in early-stage patients, as were the majority of patients in our cohort [62]. Speech in Parkinson’s disease is affected by motor symptoms that impair the ability to produce clear and modulated speech [16]. According to the MDS-UPDRS, speech is characterized from a daily living aspect as soft, slurred, and uneven, and from a motor aspect as lacking modulation, clarity, and volume [63]. Consequently, we evaluated speech by focusing on softness, nasality, monoloudness, monopitch, involuntary breaks, rhythm (prosody), phonation tremor, and speech clarity (dysarthria). Handwriting in Parkinson’s disease is influenced by tremor, rigidity, and bradykinesia, affecting size, clarity, and speed [42]. According to the MDS-UPDRS, handwriting is described as small, clumsy, slow, uneven, and unclear [63]. Consequently, we evaluated handwriting by focusing on micrographia, progressive micrographia, uniformity in letter dimensions and spacing, writing tremor, and overall legibility.

The selected feature set exhibits distinctive patterns associated with Parkinsonian manifestations in speech and handwriting, serving as key indicators for disease discrimination. Parkinsonian patterns in the extracted features were evaluated through cross-correlation. We created a signal space containing 50 shifted versions of the feature frames. We then generated a correlation matrix comprising the Pearson correlation coefficients of the instances within the signal space, revealing feature interdependency patterns specific to Parkinsonian markers in writing and speech. We structured the feature correlation data into numeric CSV files, with each feature contributing 50 units in matrix size. Subsequently, we converted the CSV files into images to facilitate classification, representing a color scheme where blue signifies a correlation of 0 and yellow represents 1. For complex patterns (evenness, legibility, writing combined final and speech combined final), we created combined CSV files inspired by Apple’s latest silicon system-on-a-chip (SoC) architecture, where each segment is tailored to a specific function within a multi-process operation. This approach facilitated the compilation of specialized datasets (Table 2), comprising 34,860 images. 

### 2.4. Signal Processing and Feature Extraction

**Figure 1:**
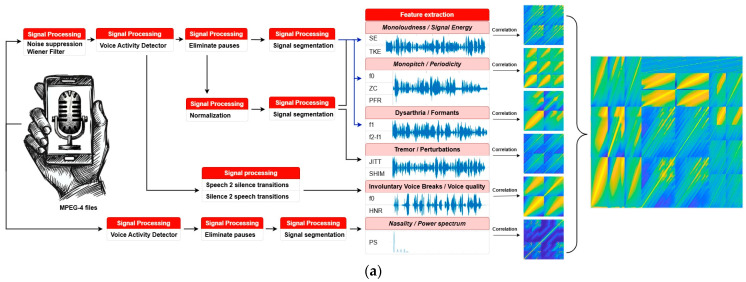

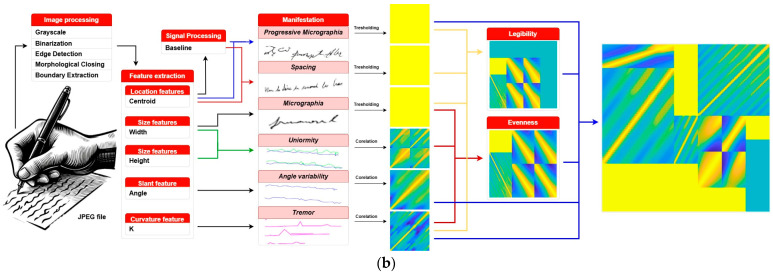
A methodological framework for the processing and assessment of: (**a**) speech and (**b**) writing.

Speech processing, Figure 1a, begins with the reduction of in-clinic noise using the Wiener optimal filter, which has been demonstrated as effective in our previous research [64] for noise reduction while preserving Parkinsonian speech features. Subsequently, an energy-based voice activity detector is used to separate speech from silence. The system then analyzes the speech for intensity, frequency, and acoustic properties, using 50 ms windows with 50% overlap for segmentation and feature extraction.

Monoloudness was analyzed using Squared Energy and Teager–Kaiser Energy to measure speech intensity variation, highlighting a limited loudness range. Monopitch analysis involved pitch, phonatory frequency range, and zero-crossing rate assessments to understand pitch variability and speech smoothness. Voice tremors were examined through jitter and shimmer measurements, indicating vocal fold vibration variability and amplitude instability, respectively. Speech articulation was evaluated by analyzing the first two formants (f1, f2), reflecting tongue movement and positioning, with reduced formant ranges indicating articulatory constraints. Hypernasality was assessed through the power spectrum, examining nasal airflow’s impact on speech resonance and quality. Involuntary voice breaks were investigated using pitch and harmonic-to-noise ratio during speech-to-silence and silence-to-speech transitions, identifying patterns of vocal instability and phonatory noise.

Handwriting analysis, Figure 1b, begins with a detailed representation of the handwriting strokes, which are analyzed for their location, size, slant, and curvature. A moving average filter tracks the stroke centroids for baseline determination, used subsequently as reference for slant analysis. This involves applying the Hough transform for straight line detection, excluding short or near-horizontal lines, and then calculating the angle relative to the baseline. 

Micrographia, progressive micrographia, and spacing were evaluated as binary attributes, by applying empirical thresholds on stroke height and width relative to paper size. In contrast, uniformity, and tremor were evaluated through cross-correlation and represented by a correlation coefficient matrix. This further enabled the development of composite images for complex disease manifestations, assigning proportional areas for individual manifestations by their order of significance. Unevenness in Parkinsonian handwriting, marked by irregularities in letter size, shape, and spacing [65], was evaluated by micrographia, nonuniformity, and the presence of tremor. Legibility was evaluated, adhering to the Handwriting Legibility Scale [66], by assessing micrographia, spacing, tremor, and evenness [67]. Ultimately, comprehensive assessment of Parkinsonian handwriting integrates appearance and legibility along progressive micrographia and tremor, constructing a composite image.

### 2.5. CNN Description

**Figure 2:**
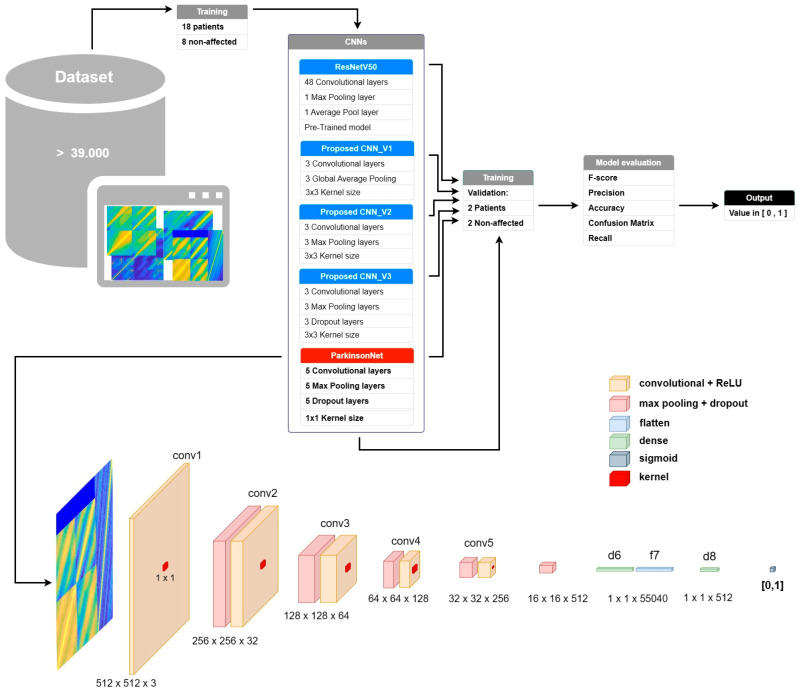
Proposed CNN workflow and architecture of ParkinsonNet.

We developed a novel convolutional neural network (CNN) architecture, Figure 2, ParkinsonNet, based on the ResNet-50_V2 architecture, to classify color correlation matrices for improved detection of Parkinson’s disease. The initial version, Proposed CNN version 1 (PCNN_V1), used three convolutional layers with global average pooling, which produced unsatisfactory results. The subsequent version, PCNN_V2, used max pooling to improve training accuracy, although this led to overfitting. To address this issue, PCNN_V3 incorporated dropout layers within each convolutional block. The final model, ParkinsonNet, integrates five convolutional layers (ranging from 32 to 512 filters), max pooling, dropout layers, a flattened layer, and two dense layers—the latter with sigmoid activation for binary classification. The model outputs a probability between 0 and 1, indicating the presence of the disease. This system was developed on an intensive computing architecture with an Intel Core i9-14900k processor (Intel Corporation, Santa Clara, CA, USA), NVIDIA GeForce RTX 4090 GPU (NVIDIA Corporation, Santa Clara, CA, USA), and 196 GB of DDR6 RAM.

We split the dataset into 60% training, 20% testing, and 20% validation data. During model validation, two individuals diagnosed with Parkinson and two healthy controls were intentionally excluded from the primary dataset, creating a separate validation cohort. We specifically selected the two patients to ensure that they exhibited collectively all the Parkinsonian manifestations under study, providing a comprehensive validation dataset. The ability of the model to generalize and predict performance in a real-life context was thoroughly evaluated through this partitioning. 

We generated visual representations of training accuracy and loss, Appendix A, from the initial training dataset. These visual graphs show the model’s performance over successive epochs, providing a clear and detailed view of the learning trajectory during the training phase. 

We evaluated the validation subset using the F1 score, a globally recognized and robust metric for binary classification [68], rather than accuracy, as is common in most published research. Whereas accuracy is a convenient indicator, it fails to differentiate between different types of errors. The F1 score on the other hand, which is expressed as the harmonic mean of precision and recall [69], serves as a definitive measure of validation accuracy, where 1 indicates ideal precision and recall, and is better suited for imbalanced class scenarios, as was the task at hand.

## 3. Results

ResNet-50_V2 was established as the performance benchmark, with the goal of developing a new CNN model that outperforms ResNet-50_V2 in both accuracy and performance. The initial iterations of our convolutional neural network (CNN), designated PCNN_V1, PCNN_V2, and PCNN_V3, displayed unsatisfactory to suboptimal performance, Table 3, achieving F1 scores as low as 66%. Our final model, ParkinsonNet, outperformed ResNet-50_V2 in the speech dataset, achieving significantly better F1 scores, Table 3, with 93.62% for articulation, 87.29% for monoloudness, 84.60% for monopitch, 93.00% for nasal speech, 93.58% for tremor, and 90.82% for involuntary breaks. When the handwriting dataset was trained, the ParkinsonNet model achieved higher F1 scores, showing 92.78% for uniformity, 90.71% for tremor, 95.65% for the combined correlation plots of evenness, and 87.93% for legibility. Micrographia, progressive micrographia, baseline, and spacing, evaluated through thresholding, resulted in an expected accuracy rate of 100%. 

ResNet-50_V2, known for its comprehensive 50-layer structure and innovative skip connections to prevent vanishing gradients, contrasts with ParkinsonNet’s design, which is meticulously crafted for classifying color correlation matrices using 1 × 1 convolutions for precise analysis. This targeted approach, combined with a streamlined five-layer architecture, ensures efficient processing tailored to healthcare needs, minimizing the requirement for vast resources and extensive training. Moreover, ParkinsonNet’s implementation of 1 × 1 max pooling layers is specifically aimed at addressing the intricacies of medical data analysis, offering a specialized solution for this field, in contrast to ResNet-50_V2’s general-purpose image recognition functionalities.

The main findings of this research show that our ParkinsonNet model achieved F1 scores of 95.74% for the speech combined final and 96.72% for the writing combined final datasets.

## 4. Discussion

### 4.1. Decoding the Results

The performance of ParkinsonNet exceeds that of ResNet-50_V2 and similar CNN models, with F1 scores of 95.74% for speech and 96.72% for writing, placing it among the top performers in literature. ParkinsonNet achieves these results on running speech, rather than isolated vowels and phonemes, and overall continuous writing appearance, rather than individual letters or repetitive strokes for writing. Our novelty lies in the embedded correlation plots for ParkinsonNet, which shift from traditional phonology/prosody-based features for speech analysis, and from plain micrographia, dynamic, and kinematic aspects for writing assessment. The results, derived from a comprehensive analysis of speech and writing data within our transdisciplinary framework and supported by our in-house developed CNN, are based on raw biomarker datasets and evaluated with transparent metrics, making them highly significant for the field. 

### 4.2. Strengths and Limitations

The research was enhanced by our transdisciplinary team approach, which brought together diverse expertise to increase its depth and breadth. The research protocol ensured data collection in a clinical setting that mirrored real-world environments using ubiquitous mobile technology. The protocol was designed to mitigate overfitting and enhance transparency using the F1 score for a more nuanced model assessment. The innovative approach to speech and writing assessment led to the creation of a substantial dataset comprising over 34,000 correlation matrices, introducing a new technique to represent complex disease patterns using composite images. Our cohort of 20 PD patients and 10 healthy controls is by no means small compared to benchmark studies in the field. In addition, the data are derived from patients who have been personally assessed by us, giving us full control over the quality of the data collection protocol.

Furthermore, we acknowledge the importance of distinguishing between Parkinson’s disease and atypical parkinsonian features, such as those observed in Progressive Supranuclear Palsy (PSP) [70] or syndromes characterized by dysautonomia [71]. PSP, for instance, is a progressive atypical parkinsonian syndrome marked by postural instability, supranuclear ophthalmoplegia, dysarthria, dysphagia, and executive dysfunction, among other features. Our research is acutely mindful of these intersecting patterns, which added complexity to our study. To explore the nuances of our focus—speech and handwriting as early PD biomarkers—within these intricate syndromes, we employed methodologies specifically designed to investigate these patterns.

### 4.3. Always Forward Thinking

Our transdisciplinary team is already developing a protocol for a prospective study on the discussed biomarkers in patients with the prodromal phase. Should promising results emerge, the creation of an open-source mobile application for monitoring could be a natural progression. The envisaged application aims to fully exploit the emerging generative AI technology, combined with real-time transmitting biosensors on users. The cross-linguistic aspects of our dataset allow for the capture of subtle variations in aspects such as articulation, prosody, and fluency, which may be shared across related language families. Consequently, this approach not only enables the detection and monitoring of PD in Romanian patients but also provides a foundation for extending our findings to other Latin, Slavic, and Germanic languages. The ultimate goal is to create a decision support system for professionals and a chatbot for users, facilitating a connection between the two through remote monitoring. For example, a personalized writing exercise therapy generated by the app and approved by the treating physician could be one of the methods to improve the quality of life for Parkinson’s patients. With a maxillofacial surgeon on our transdisciplinary team, we foresee potential upgrades to our software that could facilitate the evaluation of REM sleep behavior [72,73] disorder alongside obstructive sleep apnea. This dual assessment could be transformative in both general prevention and prodromal evaluation contexts.

### 4.4. Study Epilogue

The opening statement, “Parkinson’s Disease: A Global Challenge”, not only defines our research focus but also serves as a call to action that drives our work. The encouraging evidence from our research could inspire the academic community to build on this foundation, leading to the creation of innovative decision support systems embedded in everyday technologies. This would facilitate advances in remote monitoring and the optimization of sustainable Parkinson’s disease management. Furthermore, the impact of our findings could extend to various neurodegenerative diseases and medical fields, potentially enriching human–machine interactions, particularly in robotics.

## Figures and Tables

**Table 1:** Baseline characteristics *.

		Patients with Parkinson’s Disease (n = 20)	Healthy Controls (n = 10)
Gender	Male	12 (60%)	5 (50%)
	Female	8 (40%)	5 (50%)
Age (years)		63.9 (7.5%)	56.8 (5%)
Hoehn and Yahr Scale	1	1 (5%)	-
	1.5	4 (20%)	-
	2	2 (10%)	-
	2.5	4 (20%)	-
	3	5 (25%)	-
	4	4 (20%)	-
Subtype of Parkinson’s Disease	Akinetic-rigid	18 (90%)	-
	Tremor-dominant	2 (10%)	-

* Data are mean (SD) or n (%).

**Table 2:** The dataset size by Parkinsonian manifestation of speech and writing *.

		Patients with Parkinson’s Disease (PD)		Healthy Controls (HC)		Total Number of Images		
	Biomarkers	Number of Images	Number of Patients with Present Manifestation	Number of Images	Number of Patients with Present Manifestation	Present Manifestation	Absent Manifestation	Total
Speech	Monoloudness	2243	13 (65%)	1423	0 (0%)	1543	2123	3666
	Monopitch	2313	13 (65%)	1448	0 (0%)	1636	2125	3761
	Articulation	2243	7 (35%)	1423	0 (0%)	907	2759	3666
	Tremor	2243	11 (55%)	1423	2 (10%)	1492	2174	3666
	Involuntary voice breaks	1073	13 (65%)	460	0 (0%)	262	1271	1533
	Nasality	3059	8 (40%)	1968	1 (5%)	1433	3594	5027
	Speech (combined)	2187	20 (100%)	1312	0 (0%)	2187	1312	3499
	Total speech	15,361	-	9457	-	9460	15,358	24,818
Writing	Micrographia	-	4 (20%)	-	0 (0%)	-	-	-
	Progressive micrographia	-	7 (35%)	-	0 (0%)	-	-	-
	Baseline	-	11 (55%)	-	0 (0%)	-	-	-
	Spacing	-	2 (10%)	-	0 (0%)	-	-	-
	Angle	1100	11 (55%)	450	0 (0%)	475	1075	1550
	Uniformity	1100	15 (75%)	450	0 (0%)	615	935	1550
	Tremor	1628	9 (45%)	690	0 (0%)	663	1655	2318
	Evenness (combined)	1091	12 (60%)	450	0 (0%)	505	1036	1541
	Legibility (combined)	1092	7 (35%)	450	0 (0%)	232	1310	1542
	Writing (combined)	1092	20 (100%)	450	0 (0%)	840	701	1541
	Total writing	7103	-	2940	-	3330	6712	10,042
Total dataset		22,464		12,397		12,790	22,070	34,860

* Data are n (%).

**Table 3:** Classification performance of the Parkinsonian manifestations in speech and writing *.

Biomarkers		Convolutional Neural Networks F1 Accuracy				
		Proposed_ CNN_V1	Proposed_ CNN_V2	Proposed_ CNN_V3	ResNet-50_V2	ParkinsonNet
Speech	Articulation	83.09%	92.08%	89.43%	88.63%	**93.62%**
	Monoloudness	81.99%	84.54%	86.60%	83.25%	**87.29%**
	Monopitch	67.85%	88,18%	89.95%	75.57%	**84.60%**
	Nasal Speech	79.43%	86,00%	82.25%	83.48%	**93** **.00%**
	Tremor	71.90%	71,53%	73.51%	76.13%	**93.58%**
	Involuntary Breaks	75.18%	78.03%	82.93%	89.53%	**90.82%**
	Speech (combined)	77.92%	81.68%	84.63%	89.54%	**95.74%**
Writing	Tremor	74.82%	80.09%	89.46%	84.55%	**90.71%**
	Uniformity	69.59%	73.52%	75.48%	87.92%	**92.78%**
	Evenness	83.98%	86.37%	85.27%	89.41%	**95.65%**
	Legibility	79.46%	78.38%	79.51%	86.96%	**87.93%**
	Writing (combined)	81.42%	83.61%	85.96%	88.44%	**96.72%**

* Data are %.

**Figure A1:**
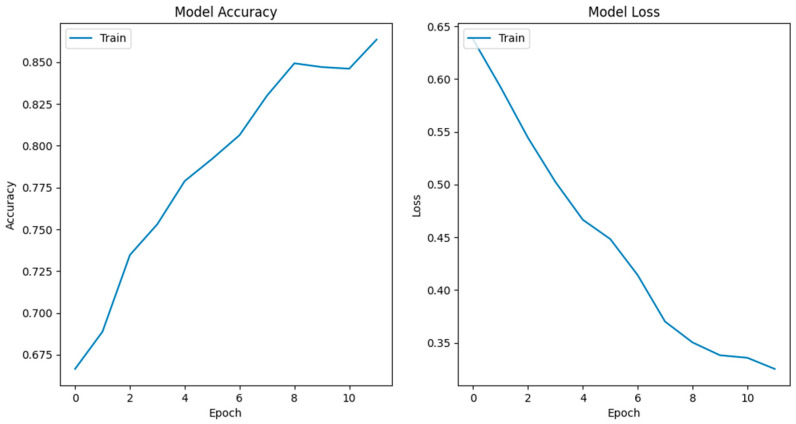
Results for ParkinsonNet accuracy and loss over epochs during training on articulation: model accuracy and model loss.

**Figure A2:**
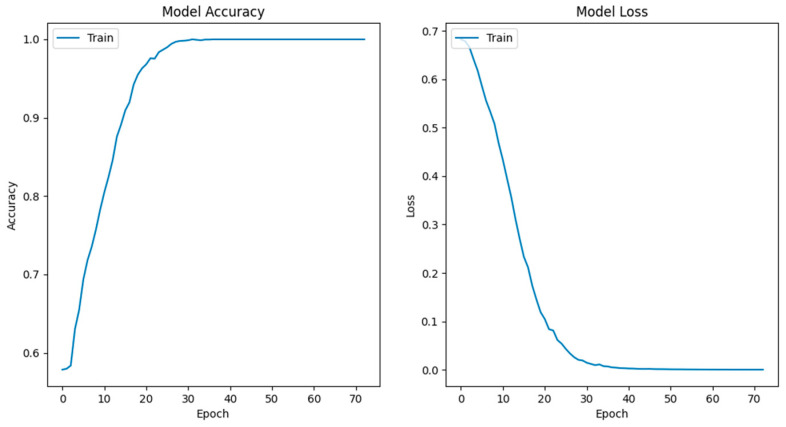
Results for ParkinsonNet accuracy and loss over epochs during training on monoloudness: model accuracy and model loss.

**Figure A3:**
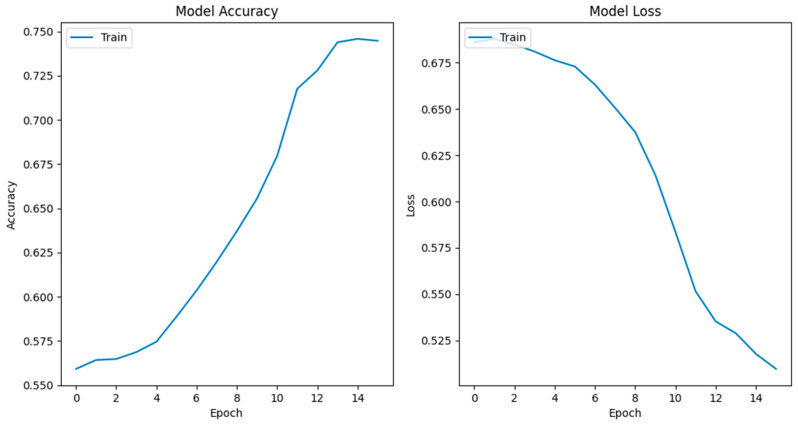
Results for ParkinsonNet accuracy and loss over epochs during training on monopitch: model accuracy and model loss.

**Figure A4:**
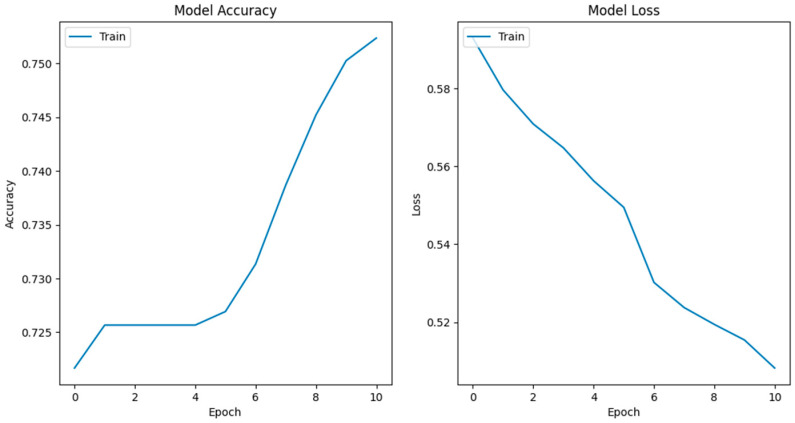
Results for ParkinsonNet accuracy and loss over epochs during training on nasal speech: model accuracy and model loss.

**Figure A5:**
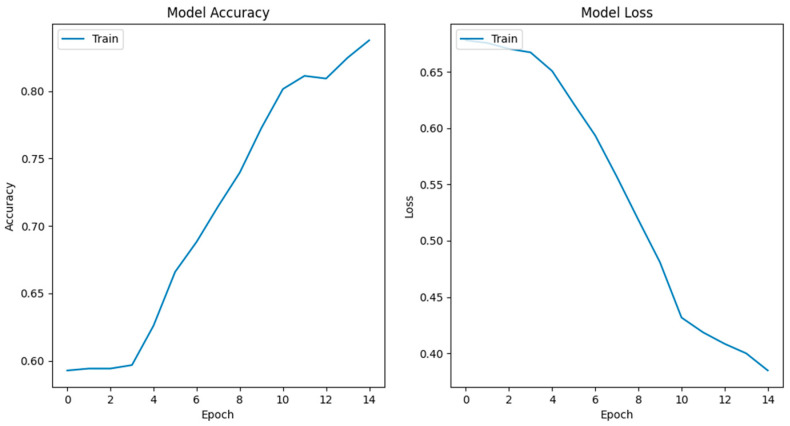
Results for ParkinsonNet accuracy and loss over epochs during training on phonation tremor: model accuracy and model loss.

**Figure A6:**
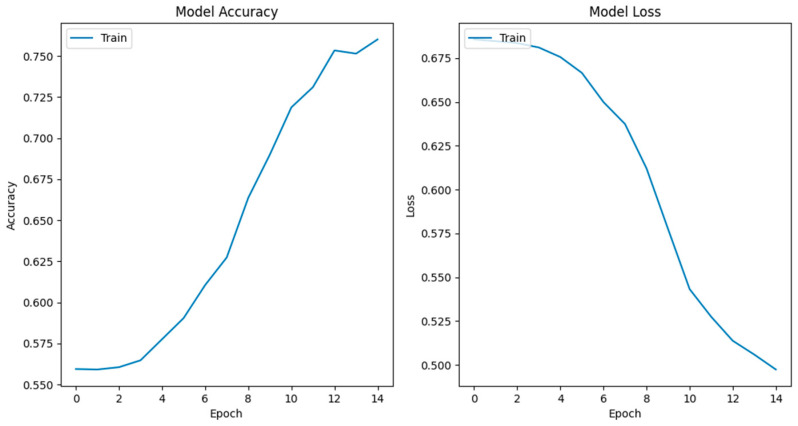
Results for ParkinsonNet accuracy and loss over epochs during training on involuntary breaks: model accuracy and model loss.

**Figure A7:**
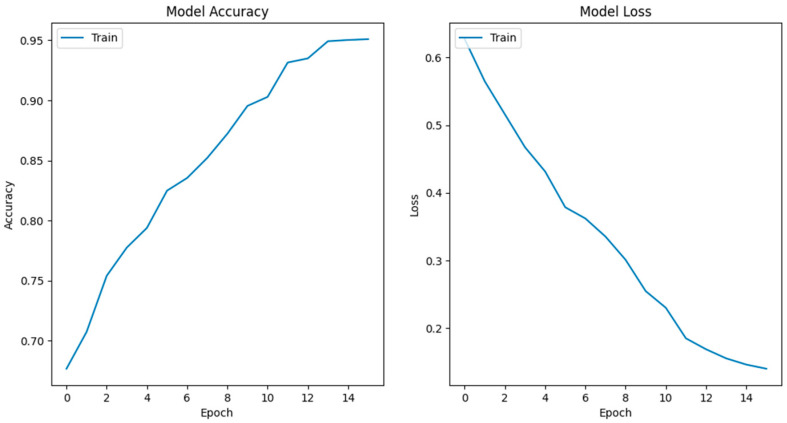
Results for ParkinsonNet accuracy and loss over epochs during training on speech combined: model accuracy and model loss.

**Figure A8:**
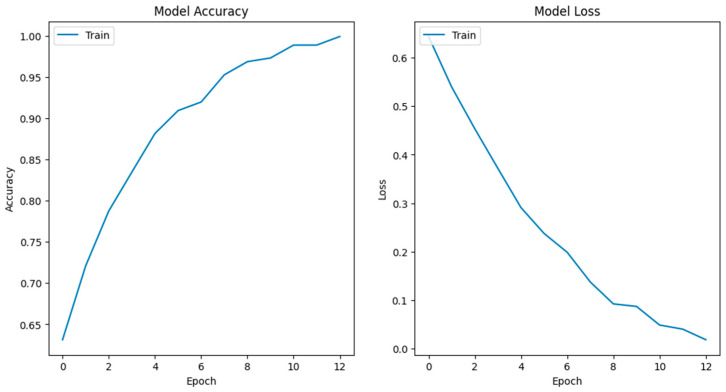
Results for ParkinsonNet accuracy and loss over epochs during training on writing tremor: model accuracy and model loss.

**Figure A9:**
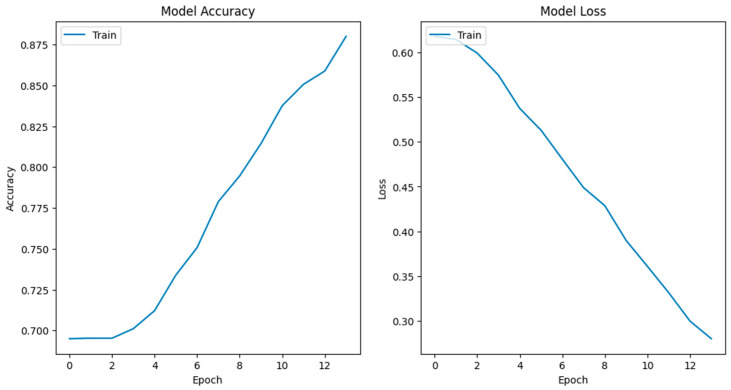
Results for ParkinsonNet accuracy and loss over epochs during training on uniformity: model accuracy and model loss.

**Figure A10:**
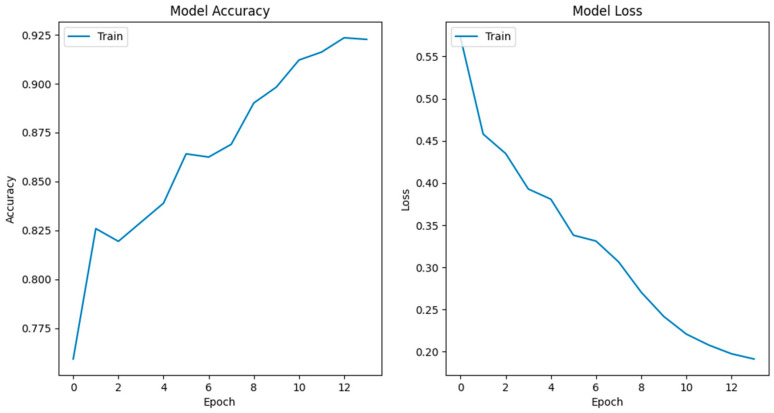
Results for ParkinsonNet accuracy and loss over epochs during training on evenness: model accuracy and model loss.

**Figure A11:**
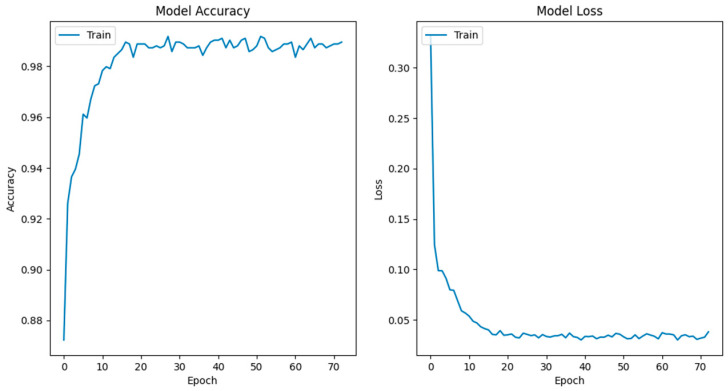
Results for ParkinsonNet accuracy and loss over epochs during training on legibility: model accuracy and model loss.

**Figure A12:**
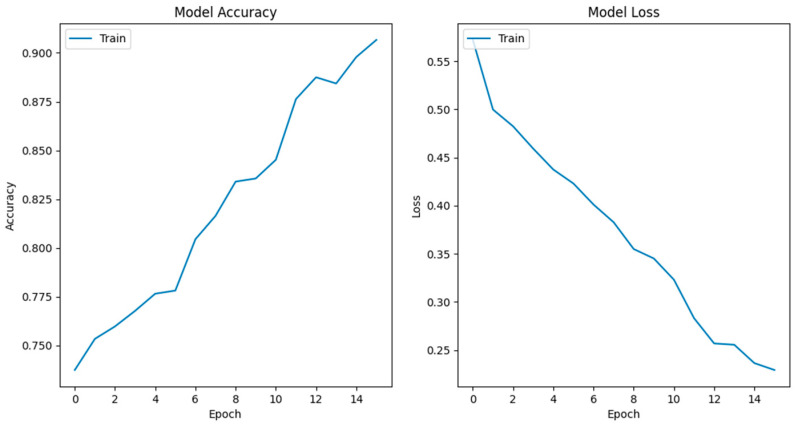
Results for ParkinsonNet accuracy and loss over epochs during training on writing combined: model accuracy and model loss.

## Data Availability

We have chosen not to make the data publicly available in accordance with the protocol statement.
